# A rise in social media use in adolescents during the COVID-19 pandemic: the French validation of the Bergen Social Media Addiction Scale in a Canadian cohort

**DOI:** 10.1186/s40359-023-01141-2

**Published:** 2023-03-31

**Authors:** Raphaël Dufort Rouleau, Carmen Beauregard, Vincent Beaudry

**Affiliations:** 1 Department of Psychiatry, Faculty of Medicine and Health Sciences, Université de Sherbrooke, Sherbrooke, Québec Canada; 2 Division of Child and Adolescent Psychiatry, Department of Psychiatry, CIUSSS de l’Estrie-CHUS (Centre Hospitalier Universitaire de Sherbrooke), Sherbrooke, Québec Canada

**Keywords:** Problematic use of social media, Social media addiction, COVID-19, Adolescent, Bergen Social Media Addiction Scale, French translation.

## Abstract

**Introduction:**

Social media use has grown dramatically since its inception in the early 2000s and has further increased during the COVID-19 pandemic. Problematic use of social media (PUSM) is a type of behavioural addiction which has generated increasing interest among mental health clinicians and scholars in the last decade. PUSM is associated with multiple psychiatric conditions and is known to interfere with patients’ daily functioning. There is no single accepted definition of PUSM, nor means of measuring it, in the literature. The Bergen Social Media Addiction Scale (BSMAS) is a helpful tool for identifying PUSM. This paper aims to validate BSMAS and to translate it from English into French, with the goal of making this clinical screening tool for PUSM available in French-language contexts.

**Method:**

This study explored the psychometric validity of the French version of the BSMAS in a sample of 247 adolescents, who were either psychiatric inpatients (the hospitalized group, n = 123) or recruited in local high schools (the community group, n = 124).

**Results:**

The adolescents in the sample reported an increase in their social media use during the COVID-19 pandemic. This increase was more pronounced in the hospitalized group. Confirmatory factorial analysis showed an excellent fit, very good internal consistency and established convergent validity for the French version of the BSMAS. A total of 15.4% of the hospitalization group and 6.5% of the community group met the recommended clinical cutoff of 24 on the BSMAS, suggesting problematic use of social media.

**Conclusions:**

The French version of BSMAS is a psychometrically validated and clinically useful tool to screen for PUSM in adolescents.

## Highlights

The Bergen Social Media Addiction Scale is valid for use in a French-speaking population.

Social media use increased more in adolescent inpatients than in community during the COVID-19 pandemic.

Adolescent inpatients should be screened for problematic use of social media.

## Background

Social media (SM) use has seen a major increase in use worldwide in the past years. According to Statista.com, as of February 2022, there are 4.65 billion users of social media worldwide, the most popular platform being Facebook©. In Canada, 96% of adolescents use SM [1]. As with other online behaviours, some adolescents are heavy users of SM, leading experts to argue that they might present a problematic use of social media (PUSM) [2]. Internet Gaming Disorder (IGD), another online behavioural addiction, has been recognized by the DSM-5-TR as a condition requiring further study to be included in the psychiatric classification [3]. There is a need for more evidence on other online behavioural addictions and their consequences on youth.

It has been estimated that 7.38% of European adolescents [4] present with PUSM. The prevalence in a large cohort of Hungarian adolescents was 4.5% [5]. Prevalence estimates might vary according to the samples studied, the classification used, and cultural and geographics factors [6]. The prevalence might also increase with the adolescents’ age [7]. Social media use has been associated with various psychiatric conditions, such as depression [8], poor sleep quality [9, 10], social anxiety [11], internalizing problems [12] and suicidal behaviours [13, 14]. There is also a relationship between IGD and PUSM, with an apparent gender difference: boys are more represented in IGD and girls are more represented in PUSM [15]. In another study, social media addiction was also weakly to moderately linked to selfitis, showing that even if both behaviours occur on social media, they are merely related and seem to reflect different addictive behaviours [16]. Evidence indicates that adolescents with poor mental health might be greater users of social media networks [17]. He et al. [18] also demonstrated that social networking site addiction was associated with potential brain structural alterations in the gray matter of the amygdala, the anterior cingulate cortex and the midcingulate cortex, which is consistent with alterations also found in other addictions. However, expected changes in nucleus accumbens were not observed, suggesting that PUSM may be distinct from other addictions. It is important to underscore that social media use also has multiple positive effects on the adolescents’ wellbeing [19, 20].

Conceptual approaches to PUSM vary greatly in the literature, as it is the case for other behavioural addictions. The component model of addiction, proposed by Griffiths [21], remains the most established. In the component model, behavioural addiction should be understood in a biopsychosocial framework, and a common etiology should be sought for all potentially addictive behaviours. He suggested a model encompassing commonalities of these conditions, including salience (the behaviour becomes the most important activity in the person’s life), mood modification (subjective consequences of engaging the behaviour), tolerance (to achieve the same effect, the activity must be engaged in for longer periods or at a greater intensity), withdrawal symptoms (such as anxiety and irritability when the activity is discontinued for a certain amount of time), conflict (interpersonal or intrapsychic) and relapse (repetition of the problematic behaviour). These components are also seen in other addiction disorders, such as the substance-use disorders. Other models exist in the field, and there are multiple scales to measure PUSM (see Cataldo et al. [22] for an extensive review). This plurality of models reflects the ever-changing nature of SM platforms, which continue to grow and adapt to the needs of the users. Some authors are critical of the status of PUSM, noting that social network sites are comprising a larger proportion of the communication tools in the modern society and cautioning against overpathologization of new behaviours. These authors suggest we should focus on SM’s negative effects without the need to classify them as true addictive disorders [23]. To consider a problematic behaviour for addictive potential, Brand et al. have suggested the use of three meta-level criteria, namely (1) scientific evidence for clinical relevance, (2) theoretical embedding and (3) empirical evidence for underlying mechanisms. There is growing evidence supporting that the above-mentioned criteria can be applied to social-network-use disorder [24].

As seen during the COVID-19 pandemic, SM use has increased in adolescents. This increase could be understood as a coping strategy used to combat the loneliness experienced during lockdowns [25], and not necessarily as an increase in the incidence of PUSM.

Aside from the conceptual framework, child and adolescent mental health professionals are confronted with youth who are struggling with behaviours related to SM, that can interfere with their functioning and healthy behaviours [26]. There is a clinical need to develop and adapt tools to screen for these youth and, ultimately, offer them resources to develop a more balanced use of social media. In this article, we aim to translate and validate the Bergen Social Media Addiction Scale (BSMAS) in a cohort of French Canadian adolescents.

## Methods

### Participants

Participants were recruited in two settings. The first group (the hospitalized group) was composed of adolescent psychiatric inpatients at the CIUSSS de l’Estrie-CHUS, Fleurimont Hospital in Sherbrooke, Quebec, Canada. The second group (the community group) was composed of adolescents recruited in four high schools in the region. Three of these were public schools and one was a private institution. Adolescents from 7th to 11th grade were recruited, and the target population was aged from 12 to 17 years old, inclusively.

### Procedure

A convenience sample was recruited from February 2021 to May 2022. Informed written consent was obtained from the adolescents and from their parent(s) or legal guardian(s). All participants were informed of the study details, the advantages and the potential risks of the study, and were advised that they were free to participate or not. The study was approved by the Ethic and Scientific committees of the CIUSSS de l’Estrie-CHUS and the Université de Sherbrooke in January 2021.

In the hospitalized group, adolescents were recruited on a specialized adolescent psychiatric ward. Adolescents of the community group were recruited after a visit by the research team to the classrooms (in person or virtually) to explain the study. After receiving the consent forms signed by the student and their parents, participants received a link to complete their questionnaire. Participants had to complete the questionnaire on LimeSurvey© V5.3.5, an online survey software. Participants who completed the questionnaire were enlisted in a draw, with prizes values which complied with the ethics guidelines of the Université de Sherbrooke.

### Measures and analysis

Socio-demographic data such as age and gender were collected. Participants were also questioned on their SM use, including the frequency of use, perceived use of SM (positive, negative or neutral), and the changes in use during the COVID-19 pandemic.

To assess PUSM, we used a French translation of Bergen Social Media Addiction Scale (BSMAS). The 6-item scale is adapted from the Bergen Facebook Addiction Scale [27] and encompasses the six domains of the component addiction model described above, rated on a five-point Likert scale ranging from 1 (very rarely) to 5 (very often). It has been translated into multiple languages [5, 28–31]. It was previously validated in a cohort of adolescents, with a threshold of 19 or more suggested to classify a youth as having PUSM, based on a latent profile analysis [5]. A recent paper suggested a cut-off of 24 to retain an “SM disorder”, based on a clinical sample of adolescents who were diagnosed by certified psychiatrists and a set of clinical criteria based on IGD found in the DSM-5 [32]. We retained a clinical cutoff of 24 in this study to suggest an adolescent had PUSM. French version of BSMAS is presented in Table 1.

**Table 1:** French version of the Bergen Social Media Addiction Scale. Échelle de dépendance aux médias sociaux de Bergen (BSMAS) – Version française *Directive* : Les questions qui suivent portent sur votre rapport aux médias sociaux (*Facebook, Twitter, Instagram* et autres plateformes semblables) et l’utilisation que vous en faites. Pour chacune d’elles, veuillez cocher la réponse qui décrit le mieux votre situation

Au cours de la dernière année, à quelle fréquence avez-vous…	Très rarement	Rarement	Parfois	Souvent	Très souvent
…passé beaucoup de temps à penser aux médias sociaux ou au moment où vous prévoyiez les utiliser?^1^	□	□	□	□	□
…ressenti un fort désir ou un besoin pressant d’utiliser de plus en plus les médias sociaux?^2^	□	□	□	□	□
…utilisé les médias sociaux pour oublier vos problèmes personnels?^3^	□	□	□	□	□
…tenté de réduire votre utilisation des médias sociaux sans y parvenir?^4^	□	□	□	□	□
…ressenti de l’agitation ou de l’inconfort s’il vous était interdit d’utiliser les médias sociaux?^5^	□	□	□	□	□
…utilisé les médias sociaux à un point tel que cela a un impact négatif sur votre travail ou vos études?^6^	□	□	□	□	□

*Note.* Aspects de la dépendance : ^1^ saillance, ^2^ envie impérieuse/tolérance, ^3^ modification de l’humeur, ^4^ rechute/perte de maîtrise, ^5^ retrait, ^6^ conflit/altération du fonctionnement. Tous les items sont notés selon l’échelle suivante : 1 (*très rarement*), 2 (*rarement*), 3 (*parfois*), 4 (*souvent*), 5 (*très souvent*)

The back-translation method of Vallerand transcultural translation [33] was performed by two independent translators. Two experts (V.B. and R.D.R.) compared the original scale with the translated one and made the necessary adjustments. The translated scale was administered to a pilot group of ten French-speaking participants to ensure that all items of the scale were understandable. Internal validity was measured using Cronbach’s α statistic.

Concurrent validity between the Bergen Social Media Addiction Scale (BSMAS), Problematic Use of Internet Questionnaire (PIUQ), *Adolescents et Substances Psychoactives* (ADOSPA) and Patient Health Questionnaire (PHQ-9) scores was assessed using correlation matrix with Spearman’s rho coefficients, with a level of statistical significance set at a p value of < 0.05. Considering the distribution of the variables, we found that Spearman’s rho rank coefficients was more appropriate. Assumptions for Pearson’s correlation coefficients are more restraining. Hence, we opted for a non-parametric coefficient and a uniform presentation. PIUQ, a 12-item scale [34] measuring Internet addiction, translated into French [35] and validated in an adolescent cohort [36], was used for concurrent validity. Problematic use of Internet can be understood as an umbrella concept encompassing multiple online behavioural addictions, including PUSM [37]. For the PIUQ in our study, the Cronbach’s α was 0.88 (95% CI [0.86–0.90]). PHQ-9 was used to measure depression level in the sample. It is a nine-item self-administered scale rated on a four-point Likert-scale ranging from 0 (never) to 3 (almost every day) that can be used to define the severity of the depression (minimal to severe) [38]. Depression is related to PUSM [15] and hence can be viewed as an indicator of concurrent validity. For the PHQ-9, the Cronbach’s α was 0.93 (95% CI [0.91–0.94]). ADOSPA is a French translation of the CRAFFT questionnaire [39]. It includes six questions, with one point given for each positive answer. A cut-off score of 2 or more indicates a risk of substance use disorder. It is used here as a marker of substance abuse, which has been associated with PUSM [40]. For the ADOSPA, the Cronbach’s α was 0.83 (95% CI [0.77–0.87]).

Part of the sample (n = 101) answered questions regarding their perception of their own use of SM and the impact on their functioning, relationships and behaviours. Items were based on the Deba-Internet Scale [41] and used to document the self-perceived consequences on participants from their PUSM.

For construct validity analysis, a confirmatory factor analysis (CFA) was performed. A series of parameters assessing goodness of fit were obtained. Chi-square test with its degree of freedom (χ^2^/df), with non-significance at p > 0.05 was used, as it is more sensitive for larger samples [42]. Other indices used were the Comparative fit index (CFI) and Tucker-Lewis indices (TLI), with cut-off value of more than 0.9 suggesting an acceptable level of goodness of fit between model and date [43]. Root mean square error of approximation (RMSEA) with 95% confidence interval (CI) and the standardized root mean square residual (SRMR) are also provided, with a satisfactory fit indicated by a value of 0.08 or less [44]. Measurement invariance (MI) across gender was examined. MI for age was not included as the age range was limited in a sample of adolescents. We tested for configural, metric and scalar invariance. There is a debate as which fit statistics should be used to measure fitness of the MI, but the majority of experts recommend comparing the fit of two nested models by computing the difference between fit indices, with change criteria as follow: ∆ χ^2^ (having the same restriction as stated above concerning larger sample), ∆ CFI < 0.01, ∆ SRMR < 0.030 (for metric invariance) and < 0.015 (for scalar invariance), and ∆ RMSEA < 0.015 [45].

Convergent validity was assessed by computing the average variance extracted (AVE), using a cutoff of 0.5 [46], and with Composite reliability (CR), with a threshold of 0.6 [46]. Standard error of measurement was computed, with an acceptable cut-off of < SD/2. Statistical analysis were performed using IBM SPSS V28 and R V.4.05, using lavaan, bluegrafir and semTools packages.

## Results

### Descriptive statistics

A total of 247 adolescents participated in the study, from which 123 were from the hospitalization group (HG; 49.8%) and 124 from the community group (CG; 50.2%). Pertinent demographic and social media use data are presented in Table 2 pertaining to the hospitalized, community and total sample. 97.6% of the cohort used at least one social media platform (96.0% for CG and 99.2% for HG), which is similar to the Canadian prevalence of use [1]. When looking at power analysis, with a total sample size of 247 participants, we obtain a 41:1 subject to item ratio regarding core factors for BSMAS, which is well above the recommended minimum 20:1 ratio for computing confirmatory factor analysis [47].

**Table 2:** Descriptive results on demographics, social media (SM) use and Bergen Social Media Addiction Scale (BSMAS) scores, with respect to hospitalized, community and total samples

Variable	Hospitalized		Community		T or χ^2^*(p-value)*	Total sample	
	Absolute	%	Absolute	%		Absolute	%
*Age (mean ; SD)*	(15.02 ; 1.42)		(14.51 ; 1.72)		0.012	(14.76 ; 1.60)	
*Sex*							
Male Female	19 104	15.4 84.6	40 84	32,3 67,7	0,002	59 188	23.9 76.1
* h(s) a day on SM*							
< 2 2–4 > 4	27 43 52	22.1 35.2 42.3	52 38 29	43.7 31.9 24.4	0.001	79 81 81	32.8 33.6 33.6
*Self-perception of SM use*							
Positive Negative Neutral	51 15 56	41.8 12.3 45.9	61 12 46	51.3 10.1 38.7	0.338	112 27 102	46.5 11.2 42.3
*Perceived change in use of SM during COVID-19 pandemic*							
Increase Decrease Same	103 6 13	84.4 4.9 10.7	87 2 30	73.1 1.7 25.2	0.005	190 8 43	78.8 3.3 17.8
*BSMAS scores*							
< 24 24 or more (PUSM)	104 19	84.6 15.4	116 8	93.5 6.5	0.023	220 27	89.1 10.9

Question on SM were answered only by those who were using at least one SM platform (n = 241)

SD: standard deviation

PUSM: problematic use of social media

### Construct and internal validity

Internal consistency of the scale was very good (Cronbach’s α = 0.84; 95% CI [0.80–0.87]). As expected, a strong positive association was observed between BSMAS and PIUQ scores (0.773; p < 0.0001), which assess similar constructs. Moderate and weak [48] positive associations (Spearman’s Rho coefficients) were detected with PHQ-9 (0.476; p < 0.0001) and ADOSPA (0.243; p < 0.01) scores respectively, showing sufficient concurrent validity, as depression symptoms [49] and substance use [50] are known comorbidities of PUSM but not similar constructs. SM self-perceived use consequences were all at least moderately correlated to BSMAS score (Spearman’s Rho coefficients from 0.421 to 0.620), which indicated a good concurrent validity between BSMAS score and self-perceived consequences of SM use (see Table 3).

**Table 3:** Social media self-perceived consequences with respect to hospitalized, community and total samples

	Hospitalized		Community			Total sample		
Variable	Absolute	%	Absolute		%	Absolute	%	*BSMAS*
At what point social media…	Mean	SD	Mean	SD		Mean	SD	*r* ^*a*^
*…Is a problem according to your friends or lover?*	2.18	1.69	2.01		1.82	2.07	1.77	0.47*
*…is a problem according to you?*	3.22	2.38	2.86		2.12	2.98	2.21	0.62*
*…harm your relationships capacities?*	2.84	2.40	1.88		1.66	2.20	1.98	0.48*
*…harm your work or your studies?*	3.88	2.66	3.30		2.53	3.49	2.58	0.62*
*…led you to psychological problems?*	3.90	2.87	2.54		2.27	2.99	2.56	0.60*
*…led you harm yourself?*	2.44	2.53	1.70		1.79	1.95	2.09	0.42*
*…led you to make temper tantrum?*	3.08	2.69	2.24		2.18	2.52	2.39	0.51*

Scores on a scale ranging from 1 (never) to 10 (always)

^a^Spearman’s Rho correlation coefficients with Bergen Social Media Addiction Scale (BSMAS) scores. *p < 0.01

SD: Standard deviation

### Confirmatory factorial analysis

**Fig. 1:**
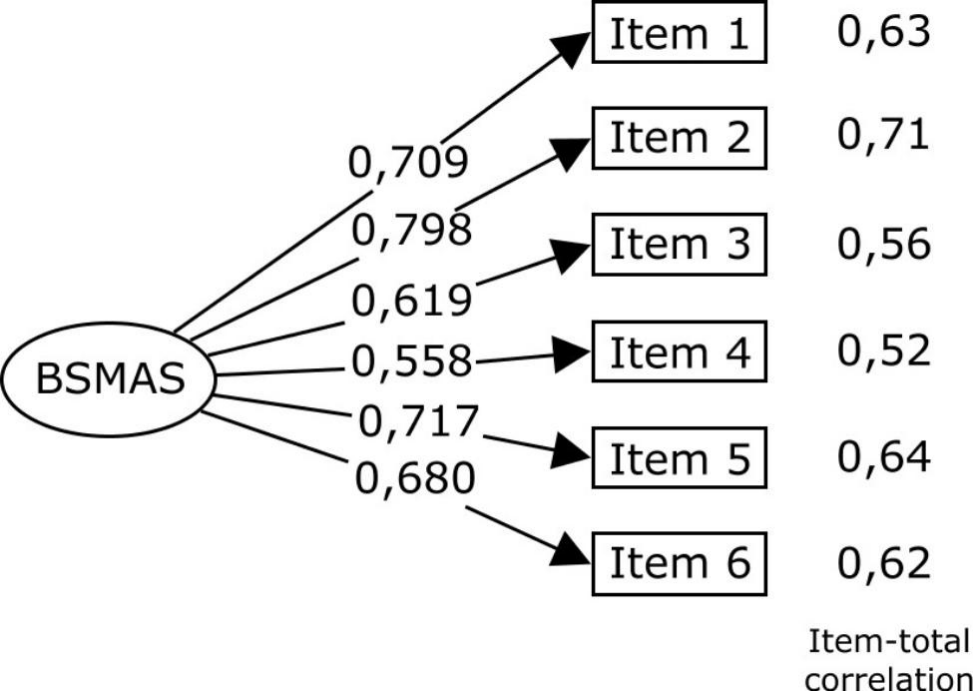
Standardized loading and Item-total correlations of the French version of the Bergen Social Media Addiction Scale (BSMAS).

Parameters of goodness of fit and reliability are presented in Table 4 and were on or above all the recommended thresholds. AVE didn’t reach the cut-off point (0.47), but CR (0.63) and Standard error of measurement (0.95) were in range of the recommended parameters. Factor loading of the model and associated item-total correlation coefficients are shown in Fig. 1. Factor loading (lambda values) were all above the recommended cutoff (> 0.50) and similar to other studies aforementioned [28–31].

**Table 4:** Confirmatory factor analysis parameters and measurement of invariance across sex for the Bergen Social Media Addiction Scale (BSMAS), French version

	χ^2^(df)	∆χ^2^(df)	CFI	∆CFI	TLI	RMSEA	∆RMSEA	SRMR	∆SRMR
BSMAS	NS	-	0.98	-	0.96	0.07	-	0.04	-
Model 1 (sex)					---				
Configural	-	26.66 (18)	-	0.98	-	-	0.06	-	0.04
Metric	-	22.74 (23)	-	1.00	-	-	0.00	-	0.04
Scalar	-	44.11 (28)	-	0.96	-	-	0.07	-	0.06

CFI: comparative fit index; TLI: Tucker-Lewis index; RMSEA: Root mean square error of approximation with 95% confidence interval (CI); SRMR: and the standardized root mean square residual

NS: non-significant

## Discussion

The objective of the present study was to validate the Bergen Social Media Addiction Scale in a cohort of French-speaking Canadian adolescents. This scale is a useful and easy-to-use tool to screen for PUSM and has been validated in multiple other languages using a similar statistical method [5, 28–31]. Confirmatory factorial analysis and invariance measurement showed a good fitness of the model, with convergent validity also demonstrating positive associations with other scales measuring associated concepts or consequences of PUSM. AVE was the only parameter not reaching the recommended threshold. This implies that the variance is explained more by measurement error than by the construct, but as the reliability parameter (CR) was reached, convergent validity of the construct can still be considered acceptable [51].

One strength of this validation is that part of the sample included psychiatric adolescent inpatients. To date and to our knowledge, most of the validation studies of the BSMAS used a sample of adolescents from the community, which diminishes generalizability of use to a clinical population. It has been shown that, in a hospitalized population, SM use in vulnerable youth was commonly associated with negative emotional experience [52], with sleep disturbance hypothesized as a mediator between negative emotional response to SM and higher symptoms severity [53]. SM use in adolescent psychiatric inpatients was also associated with greater risk of self-injurious behaviours [54]. It is worth emphasizing that, for some inpatient adolescents, SM use can also bring benefits, such as a sense of social support (from peers and family), access to positive content and access to mental health resources [55]. It is still not clear in the literature which adolescents may benefit from SM use, but we can hypothesize that adolescents with PUSM may be at an increased risk of experiencing negative effects from their SM use compared to other adolescents, as shown by the differences in BSMAS scores and consequences of use between the community and hospitalized groups.

A significant part of the sample reported an increase in their use of SM during the pandemic, which was more pronounced in the hospitalization group. This is in phase with worldwide data showing an increase in online activities during the pandemic [56]. Adolescents also experienced a dramatic increase in mental health issues and substance use [57, 58] during that period. Throughout the COVID-19 lockdowns, SM served as a substitute for face-to-face interactions and may have had protective influence against social deprivation in adolescents [59]. It is unknown if the rate of PUSM increased during the pandemic and this study was not designed to assess changes in prevalence of PUSM during this period.

As stated above, there is ongoing debate regarding the definitions and tools that should be used to evaluate PUSM in adolescents. In a rapidly evolving technological society, there is a risk of over-pathologizing adolescents who are using SM or other online services, as behavioural addictions often are time-limited and contextual [60]. Therefore, scales designed to measure PUSM, such as the BSMAS, should not be used alone in investigating problematic SM use. Rather, they should be used with caution and in combination with a thorough clinical assessment that takes into account all relevant factors of the biopsychosocial evaluation.

In comparison to other studies of validation, our sample size was modest, which may diminish the accuracy of the scale. Our study used convenience sampling, as do most studies in the field of PUSM [24], which can be a limitation when interpreting results. As aforementioned, there was a significant difference in PUSM rate between adolescents in the community group and those in the hospitalized group, but we cannot infer the direction of the relation between PUSM and psychiatric comorbidities (which are naturally over-represented in the hospitalized group).

Our study demonstrated that the French version of the Bergen Social Media Addiction Scale (BSMAS) is a psychometrically validated and clinically useful tool to screen for Problematic Use Of Social Media (PUSM) in adolescents, including adolescents hospitalized in psychiatry. Future studies should attempt to investigate PUSM risk factors and explore the relationship between psychiatric symptoms and PUSM in adolescents. Therapeutic interventions for adolescents struggling with PUSM and longitudinal evolution of these patients should also be further examined.

## Data Availability

The datasets used and/or analysed during the current study are available from the corresponding author on reasonable request.
